# Own Typology of Body Posture Based on Research Using the Diers Formetric III 4D System

**DOI:** 10.3390/jcm14020501

**Published:** 2025-01-14

**Authors:** Jacek Wilczyński

**Affiliations:** Laboratory of Posturology, Collegium Medicum, Jan Kochanowski University, Al. IX Wieków Kielc 19, 25-317 Kielce, Poland; jwilczynski@onet.pl; Tel.: +48-603-703-926

**Keywords:** new typology of body posture, thoracic kyphosis angle, lumbar lordosis angle, Diers Formetric III 4D

## Abstract

**Background/Objectives**: Body posture is developmentally variable and individually diversified. As a chain of numerous unconditioned and conditioned reflexes, it is, in its essence, a psychomotor habit. The aim of the study was to create an original typology of body posture based on measurements using the Diers Formetric III 4D system. **Methods**: The research included 303 children aged 10–12. **Results**: Taking the ranges of standards for the angle of thoracic kyphosis (42–55°) and lumbar lordosis (33–47°) into account, it was shown that there are nine types of body posture. These are as follows: reduced kyphosis, reduced lordosis (K < 42°; L < 33°); reduced kyphosis, normal lordosis (K < 42°; 33° ≤ L ≤ 47°); reduced kyphosis, increased lordosis (K < 42°; L > 47°); normal kyphosis, reduced lordosis (42° ≤ K ≤ 55°; L < 33°); normal kyphosis, normal lordosis (42° ≤ K ≤ 55; 33° ≤ L ≤ 47°); normal kyphosis, increased lordosis (42° ≤ K ≤ 55°; L > 47°); increased kyphosis, reduced lordosis (K > 55°, L < 33°); increased kyphosis, normal lordosis (K > 55°; 33° ≤ L ≤ 47°); and increased kyphosis, increased lordosis (K > 55°; L > 47°). **Conclusions**: In the final evaluation of the Diers Formetric III 4D examination, the traditional division into round, concave, round-concave, and flat backs should be supplemented and expanded to include the nine posture types mentioned above. This will enable a more precise selection of corrective exercises, which will significantly improve their quality and effectiveness.

## 1. Introduction

**Figure 1:**
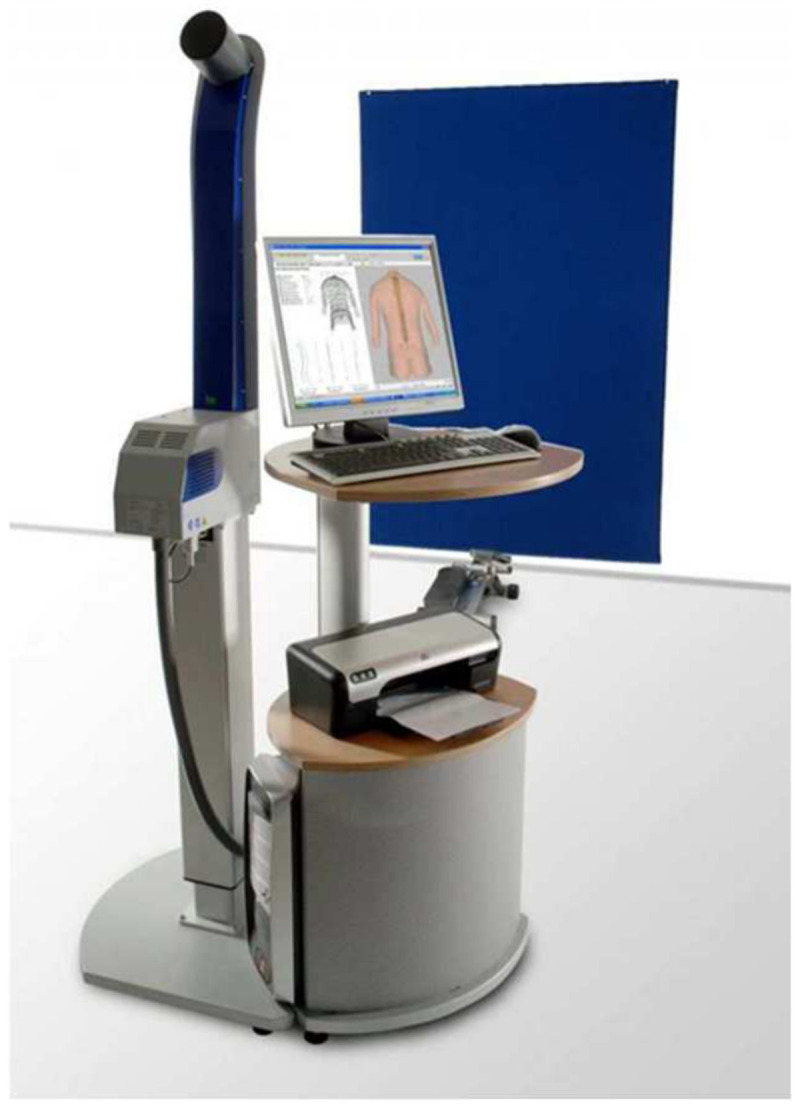
Diers Formetric III 4D system.

Body posture is developmentally variable and individually diverse. As a chain of numerous unconditioned and conditioned reflexes, it is, in its essence, a psychomotor habit. A child, adopting upright posture and developing locomotor activities, strives to master space. At the same time, he/she tries to provide the senses with the largest possible stimulus-creating area and the most perfect accuracy of stimuli reception, also wanting to create the most favourable conditions for conquering space using the motor organs. The final shaping of body posture, which is affected by the force of gravity, is determined by sets of conditioned reactions and movement habits. They are created during development on the basis of unconditioned postural, positional, support, stato-kinetic, locomotor, and many other reflexes. The psychomotor habit of body posture is a specific form of conditioned reactions within the kinaesthetic analyser, which is distinguished by a stereotyped form of movement and can constitute conscious motor behaviour or play the role of a signal for other stereotyped motor reactions. The starting point of this habit comprises stereotypical movement structures created on the basis of innate movements with a specific content, form, rhythm, force, precision, and adaptation to the nature of objects and spatial relations. The first antigravity problems that occur during the signalling of a deficit in postural tone trigger compensatory postural habits (patterns). The greater the deficit in postural tone, the weaker the postural control and the greater the difficulties in creating correct psychomotor postural habits [1]. The first typologies of human body posture were created in the second half of the 19th century in Germany. The following typologies appeared: those of Meyer, Fischer, and Staffel. Lovett, Szlemin, Dudziński, and Haglund-Falk based their typologies on the latter. Staffel’s classification was developed into Stafford’s typology. An innovative typology was created by Brown, an American orthopaedist [2]. In Poland, there is the original body posture typology proposed by Wolański (1957, 1975), modified by Zeyland-Malawka (1992), the point method by Stobiecka (1932), the method suggested by Chrzanowska and Chojnacki (1976), and Kasperczyk’s method (1988) [2]. The development of technology has enabled the use of computers in the diagnosis and therapy of postural defects. Thanks to appropriate cards and programs, computers can perform correct analysis of posture. This eliminates time-consuming calculations and creates the possibility of the accurate and comprehensive processing of obtained images, as well as the correct documentation of each examined person. Computer methods are precise and non-invasive. Due to their high convergence with clinical and radiological examinations, they allow for the elimination of some unnecessary and health-affecting X-ray examinations and more frequent objective monitoring of people affected by postural defects. In the diagnosis of postural defects, methods such as projection moiré, ISIS, Posturometr-S, Metrecom System, the thermovision method, and Spine 3D [3] are used. One of the methods applied for assessing body posture that appeared not long ago is the Diers Formetric III 4D optoelectronic system (Figure 1). It is currently the most cutting-edge, non-invasive posture assessment technology [4,5,6,7,8,9]. Body posture tests using the Diers Formetric III 4D method have been conducted since 2010 at the Posturology Laboratory at UJK Collegium Medicum in Kielce. It was then that the first Diers Formetric III 4D system in our country was purchased. To date, over ten thousand people have been examined. The traditional division of posture should be supplemented and expanded. The aim of this research was to create an original typology of body posture based on measurements using the Diers Formetric III 4D system.

## 2. Materials and Methods

The study included 303 children aged 10–12. The total number of girls under study was 143 (47.18%) while the number of boys totalled 160 (52.82%). The largest group consisted of 10-year-old children, equalling 133 participants (43.85%). Among them were 66 girls (49.24%) and 67 boys (50.76%). There were 107 11-year-old children (35.55%). There were 47 girls (43.93%) and 61 boys (56.07%). The smallest group comprised 12-year-old children, with a total of 62 individuals (20.60%).

Among them were 30 girls (48.39%) and 32 boys (51.61%). The χ^2^ test showed that the distribution of boys and girls did not significantly differ in terms of age (*p* = 0.69) (Table 1). Before initiating the study, the children and their parents were informed about the purpose, course, and duration of the study. All parents provided their written informed consent for their child to participate in the study. The study was conducted in 2016 in the Laboratory of Posturology at UJK in Kielce. The selection of the subjects was random and was conducted according to the principle of randomisation after establishing the criteria that the individual groups should meet. All research procedures were performed in accordance with the applicable 1964 Declaration of Helsinki and with the consent of the University Bioethics Committee for Scientific Research at the Jan Kochanowski University in Kielce (Resolution No. 5/2015).

### 2.1. Examination of Body Posture Using Diers Formetric III 4D Method

**Figure 2:**
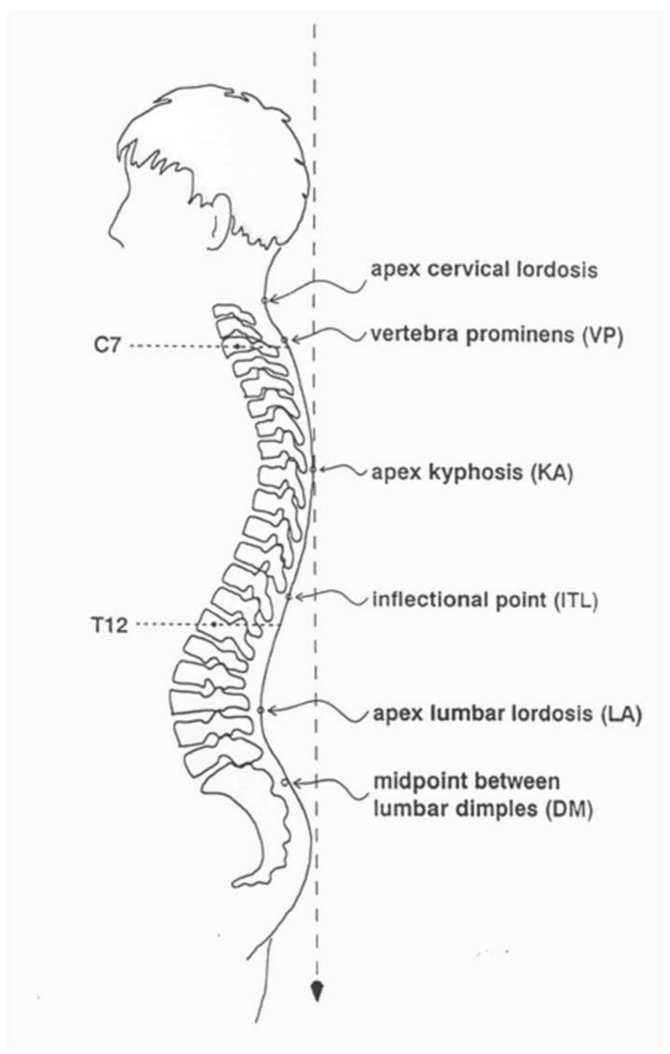
Fixed anatomical points in the sagittal plane—‘Average’ Diers Formetric III 4D analysis.

**Figure 3:**
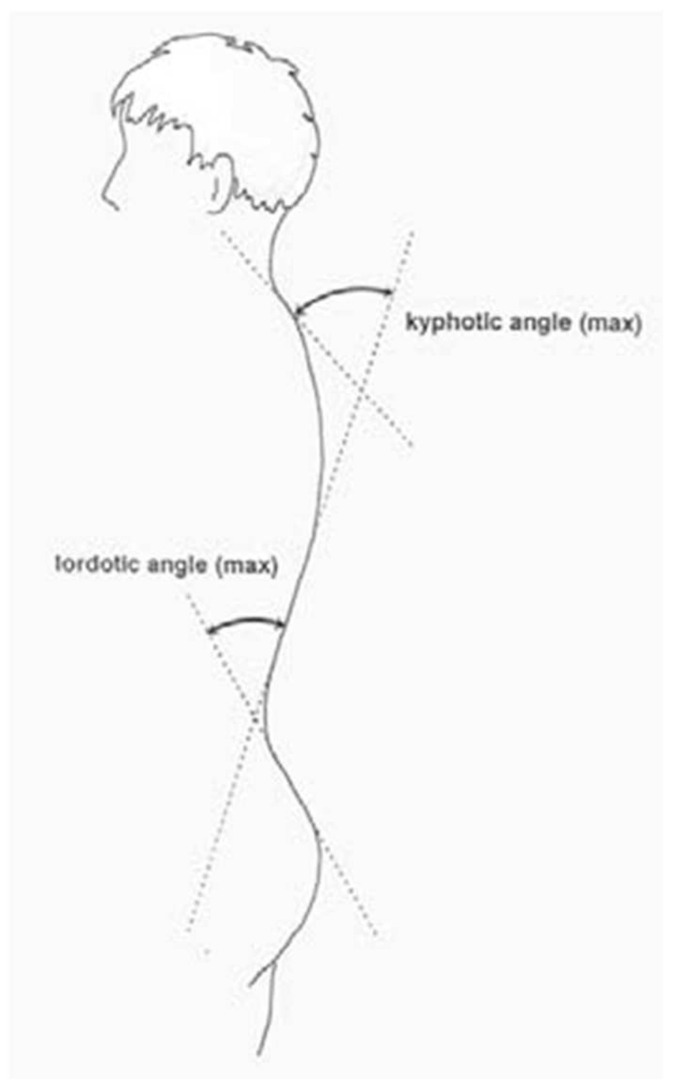
Method of determining angle of thoracic kyphosis and lumbar lordosis angles LA—‘Average’ 4D Diers Formetric III 4D analysis.

Body posture was assessed using the Diers Formetric III 4D optoelectronic system. This is a photogrammetric video recording using the process of raster stereography. Based on the obtained data, a precise three-dimensional model of the back surface is created. An important feature of this device is the spatial analysis of the body [10,11,12,13,14,15,16]. Taking the anatomical and biomechanical assumptions of the model into account, it allows for the calculation of fixed anatomical points, spinal curves, e.g., the midline of the spine and the rotational curve, as well as the parameters of the spatial form of the back resulting from these calculations. The Diers Formetric III 4D optoelectronic method allows for fast, radiation-free, and large-area optical measurement of body posture. Using this system, various clinical issues related to the objective and quantitative analysis of body posture can be presented [17,18,19,20,21,22,23]. Based on the achieved data, a precise three-dimensional model of the body surface was created [24,25,26,27,28,29]. The room in which the body posture examination was performed was darkened so that sunlight did not fall directly on the body. The subject, undressed to shorts, stood with his/her back two metres away from the device, consisting of a digital video camera and a projector. The projector emitted parallel measurement lines onto the surface of the back, and the digital video camera transmitted a three-dimensional image to the computer [30,31,32,33,34,35,36,37,38,39]. The examination was conducted via the DiCAM program using the ‘Average measurement’ mode, which consists of taking a sequence of 12 photos, which, by creating an average value, reduces the variances of posture and thus improve the clinical value of the examination. The following variables were analysed:

- The inflexion point ICT (mm), which is the cervical-thoracic inflexion point, i.e., the point of the highest surface inclination in the cervical spine (above the kyphotic apex),
- The kyphotic apex KA (VPDM) (mm), which is the posterior apex of the sagittal profile in the thoracic spine,
- The inflexion point ITL (mm), which is the thoracolumbar inflexion point, i.e., the point of the highest negative surface inclination in the area between the kyphotic and lordotic apexes,
- The lordotic apex LA (VPDM) (mm), which is the anterior apex of the sagittal profile in the lumbar spine (Figure 2),
- The inflexion point ILS (mm), which is the lumbar-sacral inflexion point, i.e., the point of the highest positive slope of the surface between the lordotic apex and sacral kyphosis,
- The kyphotic angle ICT-ITL (max) (°), which is the maximum angle of kyphosis, measured between the tangents to the surface of the upper inflexion point ICT near VP and the thoracolumbar inflexion point ITL,
- The kyphotic angle VP-ITL (°) is the kyphosis angle measured between VP and the thoracolumbar inflexion point ITL,
- The lordotic angle ICT-ITL (max), which is the maximum lordosis angle measured between the tangents to the surface of the thoracolumbar inflexion point ITL and the lower lumbar-sacral inflexion point ILS,
- The lordotic angle ITL-DM (°), which is the angle of lordosis measured between the tangents to the surface of the lumbar-sacral inflexion point ITL and DM (Figure 3). The reliability of body posture and spinal analysis using the Diers Formetric III 4D was confirmed by comparison with digital and numerically analysed X-ray images. The results of body posture measurement were based on the standards created by Harzman [40]. The values of 42° ≤ K ≤ 55°; 33° ≤ L ≤ 47° [41] were adopted as the standard for the angles of thoracic kyphosis and lumbar lordosis.

### 2.2. Methods of Statistical Analysis

The variables were verified for normality of distribution using the Kolmogorov–Smirnov and the Shapiro–Wilk tests. Depending on the distribution compliance of scale type variables with normal distribution, and the values of skewness and kurtosis, parametric or non-parametric tests were used. For qualitative and discrete variables, the distributions of counts and percentages were calculated.

The age distributions for boys and girls were tested using the χ^2^ test of independence. The Student’s *t*-test and one-way ANOVA were applied to determine the differences in body posture variables between girls and boys. Fractions (%) in relation to the types of postural defects among girls and boys were compared using the structure index test. For analysis of power justifying the sample size, the formula for sample size for the Z test from two proportions was used. The level of statistical significance was assumed as *p* < 0.05.

## 3. Results

In the whole group, the mean value of the inflexion point ICT (mm) was −0.149 and the standard deviation totalled 9.955. The kyphotic apex KA (VPDM) (mm) was, on average, −131.570 and the standard deviation was 27.833 mm. The inflexion point ITL (mm) demonstrated a mean value of −233.089 and the standard deviation was 37.653. The mean value of the lordotic apex LA (VPDM) (mm) was −307.206 and the standard deviation equalled 33.359. The inflexion point ILS (mm) was at a mean value of −372.207 with a standard deviation of 32.472. The mean value of the kyphosis angle ICT-ITL (max) (°) was 43.348 with the standard deviation being 9.406. The mean kyphosis angle VP-ITL (°) was 40.316 and a standard deviation of 9.751. The lordosis angle ITL-ITS (max) (°) totalled 40.262, on average, with a standard deviation of 9.321. The ITL-DM lordosis angle (°) was 36.654, on average, and the standard deviation was 9.163 (Table 2).

In the group of girls, the mean ICT inflexion point (mm) was 1.562 with a standard deviation of 9.922. The mean kyphosis apex KA (VPDM) (mm) totalled −126.348 with a standard deviation of 30.352. The mean ITL inflexion point (mm) equalled −228.560, and the standard deviation was 37.802. The mean lordosis apex LA (VPDM) (mm) was −304.258 with a standard deviation of 34.162. The inflexion point ILS (mm) value was −371.211, with the standard deviation totalling 33.523. The mean kyphosis angle ICT-ITL (max) (°) was 41.781 with a standard deviation of 9.210. The mean value of the kyphosis angle VP-ITL (°) was 38.757 and the standard deviation was 9.332. The lordosis angle ITL-ITS (max) (°) was, on average, 41.870, and the deviation was 9.177. The mean value of the lordosis angle ITL-DM (°) equalled 38.145, with a standard deviation of 9.032 (Table 3).

In the group of boys, the mean inflexion point ICT (mm) was −1.678, with a standard deviation of 9.764. The kyphotic apex (VPDM) (mm) was −136.223 and the standard deviation totalled 24.543. The mean inflexion point ITL (mm) was −237.133 and the standard deviation equalled 37.172. The mean value of the lordotic apex (VPDM) (mm) was −309.838 and the standard deviation was 32.506. The inflexion point ILS (mm) was, on average, −373.096, and the standard deviation was 31.583. The mean kyphosis angle ICT-ITL (max) (°) was 44.748, with a standard deviation of 9.388. The mean value of the kyphosis angle VP-ITL (°) was 41.708 and the standard deviation totalled 9.935. The lordosis angle ITL-ITS (max) (°) was at an average of 38.827 and the standard deviation equalled 9.241. The mean value of the lordosis angle ITL-DM (°) was 35.322 and the standard deviation was 9.102 (Table 4).

Significant differences between girls and boys were found for the following variables: the ICT inflexion point (mm) (*p* = 0.00464), the KA peak kyphosis (VPDM) (mm) (*p* = 0.00198), the ITL inflexion point (mm) (*p* = 0.04847), the ICT-ITL kyphosis angle (max) (°) (*p* = 0.00610), the VP-ITL kyphosis angle (°) (*p* = 0.00855), the ITL-ITS lordosis angle (max) (°) (*p* = 0.00453), and the ITL-DM lordosis angle (°) (*p* = 0.00742) (Table 5).

Taking the norm ranges for the angle of thoracic kyphosis (42–55°) and lumbar lordosis (33–47°) into account, it was shown that there are nine types of body posture: reduced kyphosis, reduced lordosis (K < 42°; L < 33°); reduced kyphosis, normal lordosis (K < 42°; 33° ≤ L ≤ 47°); reduced kyphosis, increased lordosis (K < 42°; L > 47°); normal kyphosis, reduced lordosis (42°≤ K ≤ 55°; L < 33°); normal kyphosis, normal lordosis (42°≤ K ≤ 55; 33°≤ L ≤ 47°); normal kyphosis, increased lordosis (42° ≤ K ≤ 55°; L > 47°); increased kyphosis, decreased lordosis (K > 55°, L < 33°); increased kyphosis, normal lordosis (K > 55°; 33°≤ L ≤ 47°); and increased kyphosis, increased lordosis (K > 55 °; L > 47°) (Table 6). Considering the possibility of generalisation in the grouping of normative values of the angle of thoracic kyphosis and lumbar lordosis, a statistical method based on aggregation algorithms, such as hierarchical grouping, was used. This method allowed us to reduce all types of body posture to two main classes, which indicates the existence of two dominant patterns in the typology of body posture.

In the group of 143 girls, the majority, 41 (28.87%), demonstrated reduced kyphosis and normal lordosis, as well as normal posture with the correct course of physiological spinal curvatures at 36 (25.35%). Reduced kyphosis and reduced lordosis were present in 22 (15.49%) of the examined girls. Normal kyphosis and increased lordosis were observed in 18 (12.68%) girls. Reduced kyphosis and increased lordosis were noted in 12 (8.45%) of the examined girls. Increased kyphosis and increased lordosis were found in 10 (7.04%) girls. Increased kyphosis and normal lordosis were present in two girls (1.41%), and one person (0.7%) had normal kyphosis and reduced lordosis. Increased kyphosis and reduced lordosis were also seen in one (0.7%) individual (Table 6). Among the 160 boys, postures with the correct course of physiological spinal curvatures were dominant, i.e., 51 (32.08%). The number of respondents with reduced kyphosis and reduced lordosis as well as reduced kyphosis and normal lordosis was 26 (16.35%). There were 17 boys with normal kyphosis and increased lordosis (10.69%), 16 having normal kyphosis and reduced lordosis (10.06%), 11 demonstrating increased kyphosis and increased lordosis (6.92%) and 10 presenting increased kyphosis and normal lordosis (6.29%). There were two boys showing reduced kyphosis and increased lordosis (1.26%). Only one (1.66%) person exhibited increased kyphosis and decreased lordosis (Table 7). In the entire group of 303 children, the majority demonstrated posture with the correct course of physiological spinal curvatures, with a total of 87 (28.9%). Furthermore, 67 (22.26%) showed decreased kyphosis and normal lordosis, while 48 (15.95) individuals exhibited decreased kyphosis and decreased lordosis, 35 (11.63%) had correct kyphosis and increased lordosis, and for 21 (6.98%) children, increased kyphosis and increased lordosis were noted. In 17 (5.65%) patients, normal kyphosis and decreased lordosis were observed, and 14 (4.65%) showed signs of decreased kyphosis and increased lordosis. Increased kyphosis and normal lordosis were present in 12 (3.99%) children. Only two (0.66%) had increased kyphosis and decreased lordosis. Significant differences between the girls and boys were noted in decreased kyphosis and normal lordosis (*p* = 0.00914), decreased kyphosis and increased lordosis (*p* = 0.00309), normal kyphosis and decreased lordosis (*p* = 0.00045), as well as increased kyphosis and normal lordosis (*p* = 0.03072) (Table 7).

## 4. Discussion

Body posture is indeed genetically determined, but external environmental factors also play an important role in its formation. It is precisely environmental influences that are responsible for its developmental and intra-individual variability and, as a result, its individuality. Therefore, body posture is similar in species, but developmentally variable and individually diverse [1,42,43]. This is also evidenced by the research distinguishing its nine types. Inter-individual differences make the construction of a model of ideal posture artificial and useless. The presented original typology of body posture is based on raster stereography. As already mentioned, this is a method of optical surface measurement, in which the analysis regarding the form of the back and the reconstruction of skeletal geometry have been developed to the fullest extent as of today [1]. In order to make a correct diagnosis in the case of posture defects and especially scoliosis, X-rays are very often used in addition to clinical examination. Also, control examinations during the treatment already implemented require taking a series of X-rays at regular intervals, which is a problem from the point of view of radiation exposure. In the research conducted by the Radiation Research Department in Poland between 2012 and 2013, an alarmingly high percentage of exceedances for AP/PA chest X-rays was found in the entire age range, especially for younger children. The reason is the lack of standard procedures and practical deficiencies in meeting the requirements of Polish regulations and the EURATOM directive [44]. With the introduction of the video raster stereography (VRS) software platform by Drerup and Hierholzer, the possibility of its use in posture testing was opened up for the first time. Its application is consistent with the results obtained from X-ray images [37]. The reliability of this measurement was confirmed by comparing 478 pairs of measurements using the surface topography method and X-ray images in 113 individuals. This comparison gave a mean error (one standard deviation) of 3° vertebral rotation. This is a satisfactory result, especially since the error of radiological vertebral rotation is also 3°. When comparing the lateral deviation, a mean error of 4 mm was obtained; thus, the course of the spine is in satisfactory agreement with the radiological examination. A good result was also obtained in the comparison of pelvic obliquity and the angles of thoracic kyphosis and lumbar lordosis [45]. In this study, the intra- and inter-rater reliability of the measurement method proved to be crucial to ensure the consistency and repeatability of the results. The intra-rater reliability was confirmed by repeated measurements and was demonstrated by a high internal consistency correlation coefficient (ICC). The ICC coefficient for repeatability of measurements made by the same raters at different time periods was 0.85, which indicates very good time stability of the method. The inter-rater reliability was examined by comparing results between different raters, which also showed a high ICC coefficient, confirming the robustness of the method to subjective differences in ratings. The ICC coefficient for the results obtained by different raters was 0.78, suggesting that the method is relatively robust to subjective differences between raters.

Thanks to the application of surface topography, it is possible to detect even minor posture defects. For this reason, these measurements have a permanent position in postural diagnosis and re-education. The underlying photogrammetric measurement method is triangulation. This is a simultaneous light section process. Lines (light sections) are projected onto the back, comprising a multiple light section method, because the entire back is covered with a system of parallel lines. Furthermore, it is a simultaneous process because all light sections are recorded with a video camera in just one exposure. The exposure time is 1/25 s [45], which allows for the recording of a series of images, e.g., for the purpose of documenting a sequence of movements at short time intervals. A typical, single image of the back is the basis for evaluation and interpretation. The pattern consisting of thick and thin lines allows for the unambiguous identification of individual light sections, which is a condition for precise photogrammetric reconstruction. The deformation of individual light sections contains, together with calibration data, the overall 3D information. Structures smaller than the distance between two light sections, and therefore lying between them, are not taken into account and are, as a result, not measured. The distance between two lines on the back surface is 15 mm. Important structures, such as the C7 or lumbar fossae, are usually captured by two to three lines. The video image is then loaded into a computer, where a three-dimensional reconstruction of the back surface is created using photogrammetry and image processing methods. This produces a reconstructed back surface with about 7500 points, calculated by the interpolation of measurement points. The accuracy is in the range of 0.2 mm [46,47,48,49,50]. The reconstruction of the spine’s spatial course is possible based on the Turner–Smith model [51]. In accordance with the postulate that the rotation of the surface corresponds to the rotation of the vertebrae, those so-called flat normal point towards the vertebral centres. It is assumed that the symmetry line runs along the row of spinous processes and that the vertebrae are not deformed. In addition, the length of the vertebrae hardly changes. This can be well determined based on anatomical standards and body height. Using these conditions, the centre line of the vertebrae can be calculated and displayed as a spatial curve [51]. The results of surface topography examination showed high sensitivity and good reliability, which, however, also depends on the experience and qualifications of the person performing the examination. There is a need for further development of the Diers Formetric system. There are currently two devices of this type available on the market—Diers Formetric and Spine 3D. The advantage of the Diers system is that the examination was carried out in the DiCAM program using the ‘Average measurement’ option, which consists of taking a sequence of twelve images, which by creating an average value, reduce the variance of the posture and thus, improve the clinical value of the examination. On the other hand, the Spine 3D system takes only one image, which increases the measurement error. In the future, standards for the angle of thoracic kyphosis and lumbar lordosis should be developed separately for girls and boys, as well as separately for individual age categories. Posture is the result of many variable factors, so it is impossible and pointless to construct only one model of ideal posture. Multiple conditions mean that a significant feature of posture is its inter- and intra-individual variability. This means that the posture of each child is specific. However, this does not mean that all postures are equally good, or that they cannot be classified in any way. Therefore, we used the criteria of normal posture. In further studies, the norm regarding the angle of thoracic kyphosis and lumbar lordosis should be determined separately for girls and boys and individually for specific age categories.

## 5. Conclusions

Taking the norm ranges for the angle of thoracic kyphosis (42–55°) and lumbar lordosis (33–47°) into account, it was shown that there are nine types of body posture: reduced kyphosis, reduced lordosis (K < 42°; L < 33°); reduced kyphosis, normal lordosis (K < 42°; 33° ≤ L ≤ 47°); reduced kyphosis, increased lordosis (K < 42°; L > 47°); normal kyphosis, reduced lordosis (42° ≤ K ≤ 55°; L < 33°); normal kyphosis, normal lordosis (42° ≤ K ≤ 55; 33° ≤ L ≤ 47°); normal kyphosis, increased lordosis (42° ≤ K ≤ 55°; L > 47°); increased kyphosis, decreased lordosis (K > 55°, L < 33°); increased kyphosis, normal lordosis (K > 55°; 33° ≤ L ≤ 47°); and increased kyphosis, increased lordosis (K > 55°; L > 47°). In the final assessment of the Diers Formetric III 4D examination, the traditional division into round, concave, round-concave, and flat back should be expanded by the proposed nine types of posture. This will enable a more precise selection of corrective exercises, which will significantly improve their quality and effectiveness.

## Figures and Tables

**Table 1:** Number and sex of respondents.

Sex	Age						Total	
	10 Years		11 Years		12 Years			
	N	%	N	%	N	%	N	%
Girls	66	49.24	47	43.93	30	48.39	143	47.18
Boys	67	50.76	61	56.07	32	51.61	160	52.82
Total	133	43.85	108	35.55	62	20.60	303	100
χ^2^ = 0.716; df = 2; *p* = 0.698								

**Table 2:** Body posture variables in whole group.

Body Posture Variables	N	X	SD	Me	Min	Max	Confidence −95.00%	Confidence +95.00%
Inflexion point ICT (mm)	303	−0.149	9.955	1.98	−19.99	21.62	−1.279	0.980
Kyphotic apex KA (VPDM) (mm)	303	−131.570	27.833	−132.89	−203.67	−36.64	−134.727	−128.413
Inflexion point ITL (mm)	303	−233.089	37.653	−226.86	−353.42	−156.8	−237.359	−228.818
Lordosis apex LA (VPDM) (mm)	303	−307.206	33.359	−305.91	−425.17	−212.63	−310.989	−303.422
Inflexion point ILS (mm)	303	−372.207	32.472	−371.06	−502.49	−295.26	−375.890	−368.524
Kyphosis angle ICT-ITL (max) (°)	303	43.348	9.406	43.71	17.54	70.05	42.281	44.415
Kyphosis angle VP-ITL (°)	303	40.316	9.751	40.93	11.48	64.22	39.210	41.422
Lordosis angle ITL-ITS (max) (°)	303	40.263	9.321	40.31	9.44	66.57	39.205	41.320
Lordosis angle ITL-DM (°)	303	36.654	9.163	36.67	7.55	63.21	35.614	37.693

**Table 3:** Body posture variables in group of girls.

Body Posture Variables	N	X	SD	Me	Min	Max	Confidence −95.00%	Confidence +95.00%
Inflexion point ICT (mm)	143	1.562	9.922	4.195	−19.78	21.62	−0.084	33.208
Kyphotic apex KA (VPDM) (mm)	143	−126.348	30.352	−127.585	−202.94	−36.64	−131.383	−121.313
Inflexion point ITL (mm)	143	−228.560	37.802	−221.535	−353.42	−156.8	−234.832	−222.289
Lordotic apex LA (VPDM) (mm)	143	−304.258	34.162	−302.315	−394.33	−217.67	−309.925	−298.590
Inflexion point ILS (mm)	143	−371.211	33.523	−370.07	−454.31	−295.26	−376.773	−365.650
Kyphosis angle ICT-ITL (max) (°)	143	41.781	9.210	41.17	17.54	60.82	40.253	43.309
Kyphosis angle VP-ITL (°)	143	38.757	9.332	38.34	15.59	58.04	37.209	40.306
Lordosis angle ITL-ITS (max) (°)	143	41.870	9.177	41.635	21.57	66.57	40.347	43.392
Lordosis angle ITL-DM (°)	143	38.145	9.032	38.405	11.72	63.21	35.614	37.693

**Table 4:** Body posture variables in group of boys.

Body Posture Variables	N	X	SD	Me	Min	Max	Confidence −95.00%	Confidence +95.00%
Inflexion point ICT (mm)	160	−1.678	9.764	0.9	−19.99	16.76	−3.208	−0.149
Kyphotic apex KA (VPDM) (mm)	160	−136.233	24.543	−136.13	−203.67	−42.77	−140.078	−132.389
Inflexion point ITL (mm)	160	−237.133	37.172	−233.1	−340.19	−161.12	−242.955	−231.310
Lordotic apex LA (VPDM) (mm)	160	−309.838	32.506	−308.21	−425.17	−212.63	−314.930	−304.747
Inflexion point ILS (mm)	160	−373.096	31.583	−371.09	−502.49	−306.41	−378.044	−368.149
Kyphosis angle ICT-ITL (max) (°)	160	44.748	9.388	44.98	20.98	70.05	43.278	46.219
Kyphosis angle VP-ITL (°)	160	41.708	9.935	42.82	11.48	64.22	40.152	43.265
Lordosis angle ITL-ITS (max) (°)	160	38.827	9.241	39.17	9.44	60.66	37.380	40.275
Lordosis angle ITL-DM (°)	160	35.322	9.102	34.87	7.55	56.21	33.896	36.748

**Table 5:** Differences in body posture variables between girls and boys.

Body Posture Variables	X Girls	X Boys	Student’s *t*	df	*p*	N Girls	N Boys	SD Girls	SD Boys	F Quotient of Variance	*p* of Variance	Cohen’s *d*
Inflexion point ICT (mm)	1.562	−1.678	2.852	301	**0.00464**	143	160	9.922	9.764	1.033	0.84257	0.0114
Kyphotic apex KA (VPDM) (mm)	−126.348	−136.233	3.120	301	**0.00198**	143	160	30.352	24.543	1.529	**0.00952**	**0.1499**
Inflexion point ITL (mm)	−228.560	−237.133	1.981	301	**0.04847**	143	160	37.802	37.172	1.034	0.83535	0.01195
Lordosis apex LA (VPDM) (mm)	−304.258	−309.838	1.452	301	0.14766	143	160	34.162	32.506	1.104	0.54248	0.0350
Inflexion point ILS (mm)	−371.211	−373.096	0.502	301	0.61587	143	160	33.523	31.583	1.127	0.46539	0.04199
Kyphosis angle ICT-ITL (max) (°)	41.781	44.748	−2.762	301	**0.00610**	143	160	9.210	9.388	1.039	0.81778	0.0132
Kyphosis angle VP-ITL (°)	38.757	41.708	−2.647	301	**0.00855**	143	160	9.332	9.935	1.133	0.44823	0.0436
Lordosis angle ITL-ITS (max) (°)	41.870	38.827	2.861	301	**0.00453**	143	160	9.177	9.241	1.014	0.93518	0.0046
Lordosis angle ITL-DM (°)	38.145	35.322	2.696	301	**0.00742**	143	160	9.032	9.102	1.016	0.92698	0.00527

Statistically significant values are bolded.

**Table 6:** Types of body posture according to angle of thoracic kyphosis and lumbar lordosis.

No.	Variable	Thoracic Kyphosis Angle	Lumbar Lordosis Angle	Kyphosis and Lordosis Angles
1	Reduced kyphosis, reduced lordosis	<42°	<33°	K < 42°; L < 47°
2	Reduced kyphosis, normal lordosis	<42°	33–47°	K < 42°; 33° ≤ L ≤ 47°
3	Reduced kyphosis, Increased lordosis	<42°	>47°	K < 42°; L > 47°
4	Normal kyphosis, reduced lordosis	42–55°	<33°	42° < K ≤ 55°; L > 33°
5	Normal kyphosis, normal lordosis	42–55°	33–47°	42° ≤ K ≤ 55°; 33° ≤ L ≤ 47°
6	Normal kyphosis, increased lordosis	42–55°	>47°	42° ≤ K ≤ 55°; L > 47°
7	Increased kyphosis, reduced lordosis	>55°	<33°	K > 55°; L < 33°
8	Increased kyphosis, normal lordosis	>55°	33^–^47°	K > 55°; 33° ≤ L ≤ 47°
9	Increased kyphosis, increased lordosis	>55°	>47°	K > 55°; L > 47°

**Table 7:** Types of body posture in examined group—structure index test.

Variable	Girls		Boys		Total		Structure *p*-Index Test	Cohen’s *d*
	N	%	N	%	N	%		
Reduced kyphosis, reduced lordosis	22	15.49	26	16.35	48	15.95	0.83893	0.01168
Reduced kyphosis, normal lordosis	41	28.87	26	16.35	67	22.26	**0.00914**	**0.1507**
Reduced kyphosis, increased lordosis	12	8.45	2	1.26	14	4.65	**0.00309**	**0.17135**
Normal kyphosis, reduced lordosis	1	0.7	16	10.06	17	5.65	**0.00045**	**0.2038**
Posture with normal physiological curvatures of spine	36	25.35	51	32.08	87	28.9	0.19896	0.07395
Normal kyphosis, increased lordosis	18	12.68	17	10.69	35	11.63	0.59189	0.03083
Increased kyphosis, reduced lordosis	1	0.7	1	1.60	2	0.66	0.87637	0.00894
Increased kyphosis, normal lordosis	2	1.41	10	6.29	12	3.99	**0.03072**	**0.12471**
Increased kyphosis, increased lordosis	10	7.04	11	6.92	21	6.98	0.96637	0.0024
Total	143	47.18	160	52.82	303	100		

Statistically significant values are bolded.

## Data Availability

The data and materials supporting the conclusions of this article can be found within the article.
